# Precision medicine in mental health: applications, challenges, and recommendations

**DOI:** 10.1192/j.eurpsy.2026.12210

**Published:** 2026-04-27

**Authors:** Celso Arango, Eduard Vieta, Lourdes Fañañás, Philippe Courtet, Livia De Picker, Martien J.H. Kas, Peter Kéri, Pavel Mohr, Inez Myin-Germeys, Brenda W.J.H. Penninx, Andreas Reif, Martina Rojnic Kuzman, Marion Leboyer, Andrea Fiorillo, Ana Catalán

**Affiliations:** 1 Department of Child and Adolescent Psychiatry, Hospital Universitario La Paz, IdiPAZ, School of Medicine, Universidad Autonoma de Madrid, CIBERSAM, Madrid, Spain; 2 Department of Medicine, School of Medicine & Health Sciences, University of Barcelona (UB), Barcelona, Catalonia, Spain; 3 Bipolar and Depressive Disorders Unit, Hospital Clinic, Barcelona, Catalonia, Spain; 4 Institut d’Investigacions Biomediques August Pi i Sunyer (IDIBAPS), Barcelona, Catalonia, Spain; 5 Centro de Investigacion Biomedica en Red de Salud Mental (CIBERSAM), Instituto de Salud Carlos III, Madrid, Spain; 6 Institute of Neurosciences (UBNeuro), Barcelona, Spain; 7 Department of Evolutionary Biology, Ecology and Environmental Sciences, Faculty of Biology, University of Barcelona, Barcelona, Spain; 8 Biomedicine Institute of the University of Barcelona (IBUB), Barcelona, Spain; 9 Health Institut Carlos III, Network Centre for Biomedical Research in Mental Health (CIBER of Mental Health, CIBERSAM), Madrid, Spain; 10 Department of Emergency Psychiatry and Acute Care, Lapeyronie Hospital CHU Montpellier, France; 11 IGF, Univ. Montpellier, CNRS, INSERM, Montpellier, France; 12 Collaborative Antwerp Psychiatric Research Institute, University of Antwerp, Antwerp, Belgium; 13 University Psychiatric Hospital Campus Duffel, Duffel, Belgium; 14 European College of Neuropsychopharmacology (ECNP); 15 Groningen Institute for Evolutionary Life Sciences, University of Groningen, Groningen, the Netherlands; 16 Global Alliance of Mental Illness Advocacy Networks-Europe (GAMIAN-Europe), Brussels, Belgium; 17 Clinical Department, National Institute of Mental Health, Klecany, Czechia; 18 Third School of Medicine, Charles University, Prague, Czech Republic; 19 Center for Contextual Psychiatry, Department of Neurosciences, KU Leuven, Belgium; 20 Department of Psychiatry, Amsterdam UMC, Vrije Universiteit, the Netherlands; 21 Department of Psychiatry, Psychosomatic Medicine and Psychotherapy, University Hospital Frankfurt – Goethe University, Frankfurt am Main, Germany; 22 Fraunhofer Institute for Translational Medicine and Pharmacology (ITMP), Frankfurt am Main, Germany; 23 Zagreb School of Medicine and Zagreb University Hospital Centre, Zagreb, Croatia; 24 INSERM U955 IMRB, Translational Neuropsychiatry laboratory, AP-HP, Hopital Henri Mondor, DMU IMPACT, Paris Est Creteil University (UPEC), Fondation FondaMental, Creteil, France; 25 Department of Psychiatry, University of Campania “L. Vanvitelli”, Naples, Italy; 26 Department of Psychiatry, Integrative Mental Health Research Group, Biobizkaia Health Research Institute, Osakidetza, Basurto University Hospital, Department of Neurosciences, Campus of Leioa, University of the Basque Country (UPV/EHU), Spanish Network for Research in Mental Health (CIBERSAM), Carlos III Health Institute, Carlos III Plaza de Cruces s/n. 48903, Barakaldo, Bizkaia, Spain

**Keywords:** artificial intelligence, biomarkers, digital mental health, personalised medicine, Precision psychiatry

## Abstract

Mental disorders represent a major and growing public health challenge in Europe and worldwide, characterised by marked clinical, biological, and functional heterogeneity, that limits the effectiveness of current diagnostic and therapeutic approaches. In recent years, advances in precision medicine have initiated a paradigm shift in psychiatry, offering new opportunities to improve prevention, prediction, diagnosis, treatment selection, and long-term management by integrating biological, psychological, social, and environmental information.

This EPA Guidance Paper provides an overview of the current state of precision medicine in mental health and outlines its potential clinical, scientific, and policy implications. We review key advances in genomics, epigenetics, neuroimaging, transcriptomics, digital technologies, and artificial intelligence, highlighting their relevance across the full clinical pathway, from risk prediction and early detection to treatment personalisation and monitoring. We also examine major barriers to implementation, including limited biomarker validation, insufficient representativeness of research populations, ethical and regulatory challenges, data protection concerns, and inequalities in access across healthcare systems.

Based on the available evidence, we propose strategic recommendations to support the responsible and equitable integration of precision approaches into mental health care in Europe. These include strengthening translational research, promoting multidisciplinary collaboration, updating regulatory and ethical frameworks, enhancing professional training, and prioritising mental health within national and European research and health agendas. By addressing these challenges, precision psychiatry has the potential to contribute to more effective, person-centred, and sustainable mental health care, while supporting innovation, reducing stigma, and improving outcomes for patients and society.

## Background and rationale

One of the main challenges in mental health care is the marked clinical, biological, and functional heterogeneity of mental disorders [1]. This heterogeneity limits prognostic accuracy and constrains the development and implementation of effective interventions [1, 2]. In many cases, individuals with distinct underlying biological alterations receive the same clinical diagnosis, while similar symptoms can result from different pathophysiological mechanisms [2–7]. Consequently, patients grouped under the same diagnostic category often show highly variable responses to the same intervention, and conversely, similar responses can be observed across different diagnostic categories [1].

Over the past decade, advances in precision medicine have led to the incorporation of advanced diagnostic tools and integrated analyses of biological, genetic, psychological, environmental, and social data. This integration supports a more accurate characterisation of the aetiological bases of mental disorders and opens the door to improved prediction, prevention, diagnosis, and treatment tailoring [1, 8–10].

Although they share most principles and goals, precision approaches have some specificities in the fields of psychology and psychiatry, but this distinction may be questionable at different levels, as in both disciplines, a precision-based approach aims to use objective data to stratify patients into subgroups that share common etiopathophysiologies, have a similar prognosis, and respond to specific treatments with a given mechanism of action. In psychology, precision approaches are more focused on cognitive, affective, and behavioural processes to tailor non-pharmacological interventions to a patient’s functional and psychological profile [11, 12]. In psychiatry, precision approaches additionally support pharmacological treatment selection based on genetic, neurochemical, neurophysiological, or neuroimaging biomarkers, with the aim of optimising therapeutic response, minimising adverse effects, and considering medical comorbidities and differential diagnoses [1, 8–10].

Within this scenario, precision psychiatry emerges from the premise that even when two individuals present similar symptoms, the underlying causes may differ. This is particularly relevant in mental health due to strong influences from genetic, neurobiological, and environmental factors. The overarching objective is to improve diagnostic precision, either through the development of new diagnostic systems based on objective and measurable criteria or through improving the precision of current diagnostic frameworks, while tailoring interventions to increase efficacy, improve adherence, and anticipate response, thereby reducing outcome variability. This requires considering both intrinsic factors (e.g., genetics, neuroplasticity, individual biology) and extrinsic factors (e.g., social and family environment, lifestyle, external conditions) [13–17] as well as their interaction.

Overall, the development and implementation of precision medicine in mental health provides a robust foundation for a more comprehensive, evidence-based, and person-centred model of care, while also raising important clinical, ethical, health economic, and policy considerations that are central to contemporary mental health systems.

Although precision and personalised medicine are sometimes used indistinctively, we believe that “personalised medicine” is a broader term that includes care tailored to the individual patient, while precision psychiatry reflects an approach to care guided by measurable data to place patients into biologically or clinically meaningful subgroups.

## Applications of precision medicine in mental health

Advances in biomedical research are improving the understanding of the biological mechanisms underpinning mental disorders and are accelerating the development and validation of more precise diagnostic and therapeutic tools [1, 8].

**Figure 1.**
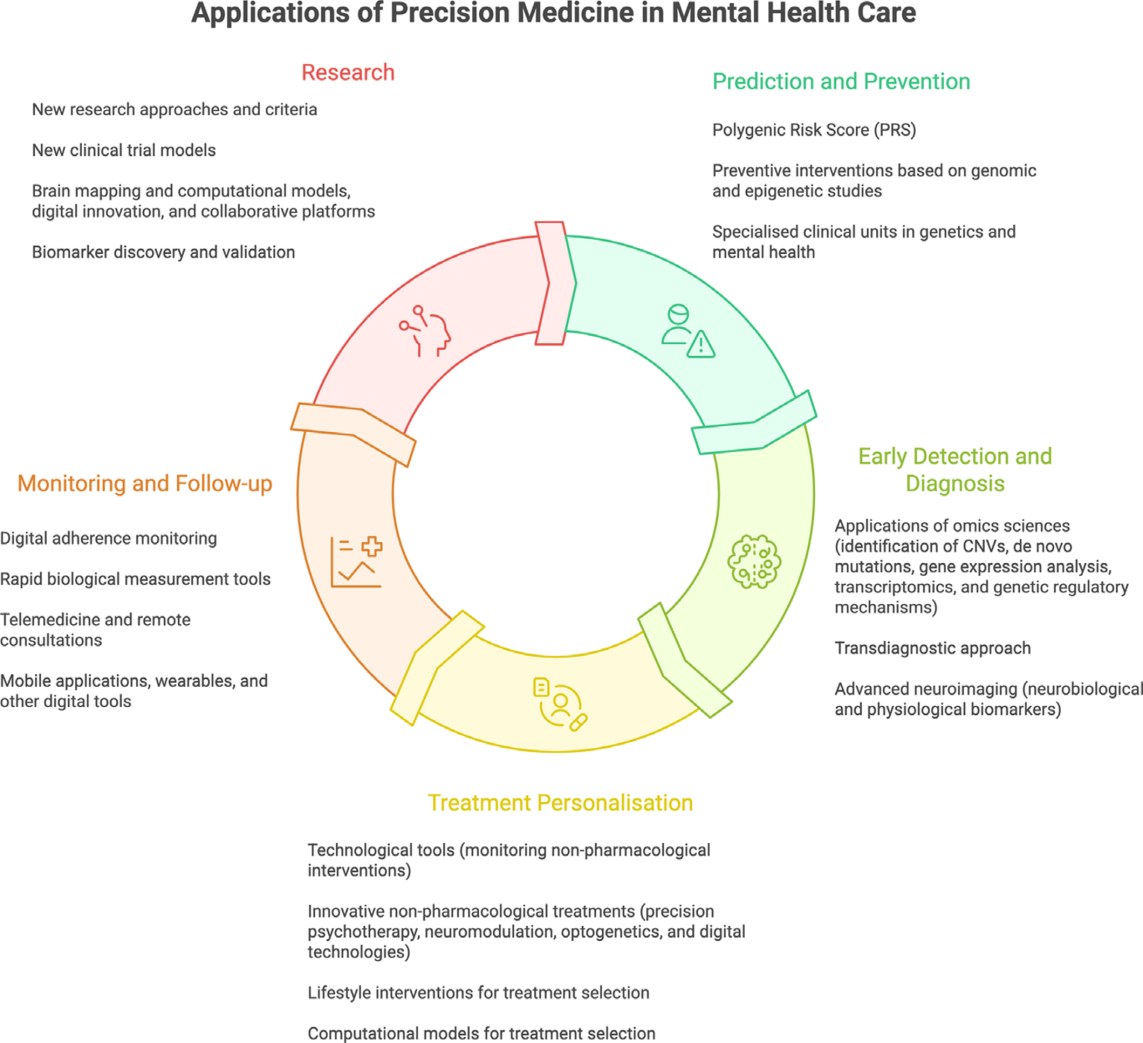
Applications of precision medicine in mental health care. This figure was created using Napkin (AI-assisted visualization tool) based on author-provided concepts and content.

In clinical care, precision medicine has potential applications across the clinical pathway, including prediction and prevention, early detection and diagnosis, treatment personalisation, and monitoring and follow-up [2]. When implemented responsibly, it can support more targeted interventions aligned with an individual’s clinical and functional profile, in a context where the societal and economic burden of mental disorders remains a major challenge for healthcare systems [15] (see Figure 1).

### Research

**Figure 2.**
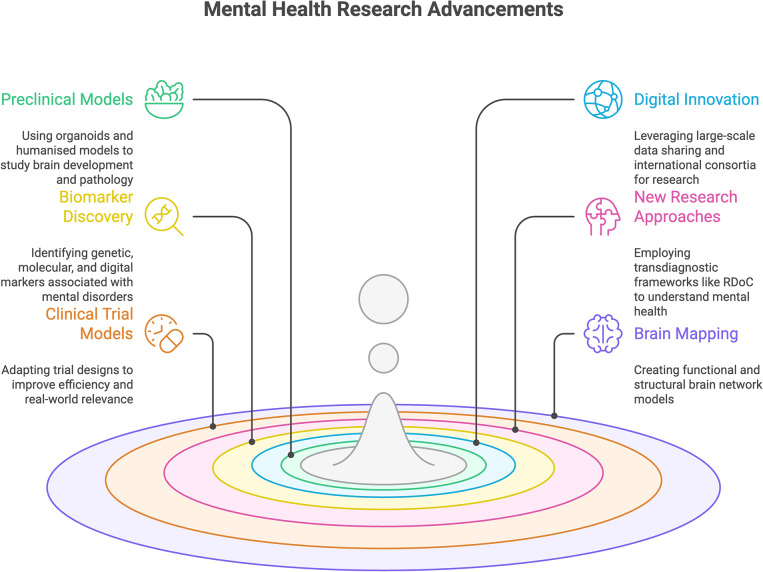
Mental health research advancements. Note: This figure was created using Napkin (AI-assisted visualization tool) based on author-provided concepts and content.

Recent advances in mental health research have generated new experimental, methodological, and collaborative approaches that underpin current developments in precision medicine (see Figure 2). These developments span research frameworks and technologies at different stages of validation and clinical translation.

#### Key research advances

- More precise preclinical studies of brain biology using human-relevant systems. Neuronal organoids and more complex brain region–specific organoids (“brain in a dish”) enable investigation of cellular and molecular mechanisms in environments closer to the human brain and facilitate evaluation of therapeutic interventions [18–20].
- Humanised animal models, for example, implanting human neurons into genetically modified mice expressing human proteins, allow study of pathophysiological processes and treatment responses in systems closer to human biology [21, 22].
- Transdiagnostic research frameworks. The RDoC (Research Domain Criteria) approach promoted by the National Institute of Mental Health studies disorders through shared symptoms, processes, and mechanisms, supporting the identification of transdiagnostic biomarkers and more homogeneous clinical subgroups, improving clinical trial precision [2–4]. Another current transdiagnostic approach is used in the HiTOP classification [23]. However, it must be stressed that both RDoC and HiTOP are meant to support research frameworks, but not to substitute clinical diagnostic systems such as DSM-5 or ICD-11.
- Expansion of transdiagnostic approaches to resistant depression, bipolar disorder, and schizophrenia, where shared mechanisms may be addressed through common strategies [5–7, 24, 25].
- New clinical trial designs. Adaptive trials enable modification of parameters (dose, treatment, sample size) based on interim results that allow patient stratification and may be useful in the context of highly variable psychiatric treatment responses [16]. N-of-1 studies evaluate treatment efficacy in single patients and support personalisation in complex cases [26]. Platform/basket trials evaluate multiple treatments across biological/clinical subgroups [27] which may aid in identifying theragnostic markers, that is biological markers that index response to a given treatment. Real‑world data and hybrid designs combine experimental evidence with digital registries and biomarkers [28]. Together, these designs support more flexible, efficient, and precise evaluation of interventions in mental health.
- Brain maps and computational modelling. Advances in neuroimaging, AI-based simulation, and genomic sequencing enable structural and functional models to understand circuits involved in cognition, emotion, and mood regulation. Initiatives such as the Human Brain Project and BRAIN Initiative support integrative neuroscience with translational potential [29, 30]. Human Cell Atlas efforts characterise brain cell types at the molecular level, enabling cellular-level insights [31].
- Biomarker discovery and validation. Consortia such as PSY-PGX [32], Psych-STRATA [33], and R-LiNK investigate genetic variants influencing treatment response, supporting biomarker targets for safer, more effective prescribing [34, 35]. Projects such as PRISM, BD2, and CIBERSAM [36] integrate neuroimaging, genetics, transcriptomics, epigenetics, and inflammation to define molecular/clinical profiles, with the aim of improving stratification and prediction of response, minimising adverse effects, and optimising adherence [24, 37–40].
- Digital innovation and collaborative platforms. EIT Health promotes digital infrastructures, AI, and clinical decision support to accelerate reproducible research, harmonise methods, and increase representativeness – crucial for precision mental health [41].
- More advanced approaches to capture the impact of the environment. Exposome research integrates all environmental risk factors into a comprehensive, poly-environmental framework [42], while the growing body of Experience Sampling Method studies enables the examination of day-to-day environmental influences on mental health [43, 44].

### Prediction and prevention

Prediction and prevention in mental health require a clear distinction between approaches that support risk stratification and those that enable actionable preventive interventions. Advances in genomic research have improved the understanding of population-level vulnerability to mental disorders, showing that most psychiatric conditions are highly polygenic and reflect the cumulative effect of multiple genetic variants with small individual effects [45]. These findings have contributed to mapping shared genetic architecture across disorders, but their direct implications for prevention remain limited.

Whole genome sequencing and genome-wide association studies (GWAS) allow aggregation of genetic variants into polygenic risk scores (PRS), which estimate relative genetic liability [46]. PRS have shown potential in research settings for schizophrenia, bipolar disorder, major depressive disorder, ADHD, and autism spectrum disorders, particularly for risk stratification and the study of shared biological pathways [45, 47]. However, their clinical utility for prevention or treatment is constrained. Current PRS explain only a modest proportion of heritability (although meaningful PRS seem to emerge, for example, schizophrenia when the sample size approaches a critical size), show reduced predictive performance outside populations of European ancestry, and do not capture rare variants or dynamic biological, environmental, and social influences [46, 48]. Importantly, as PRS are derived from conventional diagnostic assessments (mainly DSM-based categories), which carry the heterogeneity problems mentioned above, they also capture this heterogeneity. Accordingly, PRS should be considered as complementary components of an integrated risk assessment rather than standalone tools for individual-level preventive decision-making. It remains to be established whether PRS have a larger role in prediction when diagnoses are biologically oriented rather than atheoretical.

Preventive strategies in mental health rely predominantly on identifying and modifying environmental and developmental risk factors. Environmental exposures can induce epigenetic changes affecting gene expression without altering the DNA sequence, particularly during sensitive periods of development. Perinatal hypoxia, prenatal nutrition, exposure to toxins, and stress during pregnancy have all been associated with increased risk for later mental disorders [17, 49, 50]. These early-life factors represent critical windows for prevention, where timely interventions may reduce long-term vulnerability. High PRS might also identify individuals most susceptible to environmental risk in a stratified prevention paradigm.

Beyond early development, accumulating evidence highlights the influence of modifiable environmental and lifestyle factors across the life course. Research on the gut–brain axis suggests that alterations in microbiota composition are associated with depression and schizophrenia, implicating immune, metabolic, and neuroendocrine pathways in mental health risk [51]. Although causal mechanisms are still under investigation, these findings support preventive approaches targeting nutrition, physical activity, sleep, and other lifestyle-related factors [52, 53].

Effective prevention, therefore, depends on integrating biological vulnerability with psychosocial and environmental context. Concrete prevention initiatives are already emerging within European health systems; for example, specialised clinical units integrating genetics and mental health care are now being established to support clinically healthy individuals with elevated genetic risk through tailored psychoeducation, monitoring, and early psychosocial and clinical interventions [54, 55]. Such initiatives illustrate how predictive information can be translated into proportionate, non-stigmatising preventive strategies embedded within routine healthcare.

Importantly, these integrated precision approaches are also directly relevant to suicide prevention, as suicidal behaviour often emerges from the convergence of psychiatric vulnerability, biological dysregulation (e.g., stress and inflammatory pathways), and cumulative psychosocial adversity, making it a paradigmatic outcome for multilevel risk stratification and targeted preventive interventions within a precision psychiatry paradigm [56].

Overall, prediction and prevention in precision mental health require a multilevel approach that prioritises modifiable risk factors while using genomic and biological information to inform, rather than replace, preventive action. Aligning these strategies with principles of the Precision Public Health may enable earlier identification of risk, targeted preventive interventions, and a reduction in the long-term clinical, social, and economic burden of mental disorders.

### Early detection and diagnosis

Early detection is key to improving prognosis and optimising intervention effectiveness. However, current clinical practice still lacks sufficiently precise diagnostic tools for the early phases of mental disorders. Traditional classifications such as DSM-5-TR and ICD-11 are largely based on clinical observation and descriptive criteria, with limited specificity and ability to capture individual complexity, despite incremental improvements [1, 57, 58].

In response to these limitations, transdiagnostic approaches propose studying mental disorders through shared processes and mechanisms (e.g., emotion dysregulation, impulsivity, cognitive deficits) rather than isolated diagnostic entities [59]. In schizophrenia, for example, emerging approaches cluster patients using combinations of genetic, neurocognitive, and neuroimaging variables, identifying subgroups based on cognitive profiles, polygenic burden, or structural brain patterns [60]. Such work supports reconceptualising schizophrenia as a set of syndromes with shared symptoms but distinct biological mechanisms. Large consortia such as ENIGMA [61] and UK Biobank [62] integrate genetic, clinical, and biological data from large populations to refine objective and functional diagnostic criteria, facilitating biomarker discovery and the development of more precise early detection tools [63].

Within this evolving diagnostic framework, advances in omics technologies are contributing to earlier detection by identifying biological signals associated with vulnerability and early disease stages. In genomics, copy number variants (CNVs), often arising from de novo mutations, affect genes critical to neurodevelopment and are more frequent in autism spectrum disorder (ASD), ADHD, and schizophrenia. Whole genome sequencing (WGS) can detect these variants even in the absence of a family history, supporting earlier diagnosis and improved phenotype characterisation. In neurodevelopmental disorders such as intellectual disability and ASD, whole exome sequencing (WES) has demonstrated a high diagnostic yield, approaching 30–40% in specialised settings, substantially exceeding that of most diagnostic tests used in other areas of medicine. De novo mutations have also been identified in adults with intellectual disability and may relate to additional psychiatric disorders [64, 65]. Similarly, 22q11.2 deletion syndrome is associated with an increased risk of schizophrenia during adolescence, where early identification may enable preventive and tailored interventions [66].

Other molecular approaches are also being explored. Transcriptomic tools using peripheral blood mRNA are emerging, exemplified by the EDIT-B test, which combines RNA biomarkers and artificial intelligence algorithms to try to differentiate bipolar disorder from major depression, a clinically challenging but highly relevant diagnostic distinction [67, 68]. Epigenetic biomarkers are under investigation. BDNF gene methylation has been associated with mood disorders and response to antidepressant treatment (as prospectively studied in the P4D trial) [69], with hypermethylation linked to reduced BDNF protein levels, suggesting that methylation patterns may support early detection [70]. Methylome-wide association studies (MWAS) have preliminarily identified CpG methylation patterns that might be associated with major depression, including immune and inflammatory genes (e.g., MHC), and have enabled the development of methylation scores that, while not yet diagnostic, may represent progress toward blood-based epigenetic risk tools [71].

Beyond molecular markers, functional and physiological biomarkers provide complementary information relevant to early detection. Blood lipidomic profiles and neuroimaging markers [72] have shown potential utility; machine-learning models (e.g., Elastic Net regression) have identified lipid-derived patterns in individuals with dual diagnoses of substance use disorder and ADHD, with a reported diagnostic capacity of approximately 72%. Structural and functional MRI studies have identified brain volume and connectivity alterations in depression, ADHD, and ASD. In depression, distinct connectivity patterns have been described between treatment-resistant depression (TRD) and non-resistant depression (nTRD), enabling more refined stratification and potentially more personalised treatment planning [73].

Electrophysiological methods, including EEG and MEG, can detect atypical neural activity patterns relevant to neurodevelopmental risk, particularly in preterm or low-birth-weight infants [56, 57]. Biochemical markers, such as neurotransmitters, inflammatory markers, and cortisol, may also show alterations before the onset of overt clinical symptoms [58]. In addition, eye-tracking tools can identify early attentional alterations associated with ASD risk, such as declining eye contact during infancy [74].

Finally, artificial intelligence is increasingly used as an integrative adjunct to early diagnosis by detecting subtle patterns across clinical, genomic, behavioural, and neurological data, including language use, sleep patterns, and daily behaviours. Natural language processing approaches can identify signals of psychological distress in clinical interviews even when symptoms are not explicitly reported, supporting earlier identification of depression, anxiety disorders, bipolar disorder, and ASD [75–77].

### Treatment personalisation

Given heterogeneity, personalising both pharmacological and non-pharmacological treatments is a central pillar of precision mental health care. Individuals with the same diagnosis can respond differently to the same intervention; tailoring to genetic, neurobiological, psychological, and contextual features can increase efficacy and reduce adverse effects, advancing person-centred care. In this context, treatment personalisation aims to inform clinical decision-making by matching interventions to individual biological, psychological, and contextual profiles rather than relying on diagnosis alone.

#### Pharmacological treatments

A major advance in treatment personalisation is pharmacogenomics, which, in selected clinical contexts, identifies genetic profiles associated with efficacy and adverse effect risk, supporting safer and more informed prescribing decisions. Variants in cytochrome P450 genes (notably CYP2D6 and CYP2C19) influence antidepressant, antipsychotic, and mood stabiliser metabolism, determining poor/intermediate/normal/rapid/ultrarapid metaboliser phenotypes. Ultrarapid metabolisers may clear drugs before achieving therapeutic levels, while poor metabolisers may accumulate higher levels and risk-averse effects [78, 79]. Genotyping supports anticipating experience adverse reactions, optimising dosing, and selecting alternatives.

Variants affecting pharmacodynamic mechanisms are also relevant. In the serotonergic system, SLC6A4 (encoding the serotonin transporter, a target of SSRIs) polymorphisms, such as 5‑HTTLPR, influence expression and treatment response; HTR2A variants (e.g., rs6311, rs6313, rs7997012) may also influence SSRI response. Dopaminergic regulation variants, including COMT Val158Met, may affect antipsychotic response and side effects. BDNF Val66Met has also been associated with therapeutic response and clinical course. In any case, effect sizes are still too small to be individually predictive. Pharmacogenetic utility is especially relevant in complex cases with low response or unexplained side effects [80]. However, the clinical effect sizes of most pharmacodynamic variants are modest, and their utility is greatest when integrated with clinical factors rather than used in isolation.

While many associations are not yet routine, pharmacogenetic testing is increasingly available. Inclusion of pharmacogenetic analyses has been approved in EU countries in the common service portfolio (2023), including psychiatric drugs such as tricyclic antidepressants and SSRIs [81].

Novel pathways beyond the monoamines (glutamatergic, cholinergic, endocannabinoid) are under investigation for novel treatments in schizophrenia, bipolar disorder, ADHD, and ASD [82–86]. Precision approaches become particularly relevant when treatments with distinct mechanisms of action (MoA) are available (“when all you have is a hammer, everything looks like a nail”), and therefore, these advances call for precision prescribing to identify as soon as possible which patients may benefit most from a specific MoA [87].

Inflammatory biomarkers are increasingly considered for treatment stratification. Low-grade inflammation and oxidative stress have been described in depression and first-episode psychosis, including reduced glutathione and elevated IL‑6, IL‑1β, TNF‑α, and CRP levels [39, 88, 89]. N‑acetylcysteine (a glutathione precursor) has been studied for neuroprotective effects after first-episode psychosis [90]. Meta-analyses suggest that anti-inflammatory agents (e.g., celecoxib, TNF‑α inhibitors) may improve symptoms in subgroups with elevated inflammation [91]. Integration of inflammatory biomarkers supports biologically informed treatment selection [92].

#### Non‑pharmacological treatments

Precision approaches are also transforming non-pharmacological treatments. Precision psychotherapy aims to tailor interventions (CBT, psychoeducation, mindfulness) to personality, life history, context, social environment, and, when available, biological profiles. This collaborative model can improve efficacy, adherence, and patient experience. In obsessive-compulsive disorder, tailored CBT can achieve improvements comparable to pharmacological treatments [93, 94].

Neuromodulation (e.g., transcranial magnetic stimulation, deep brain stimulation) targets specific circuits implicated in treatment-resistant depression, OCD, or chronic pain and can be combined with psychotherapy or pharmacological strategies to enhance individualization [95].

Optogenetics, still experimental, combines genetic engineering with light stimulation to activate or inhibit neurons with high precision, enabling detailed study of circuits related to emotions, behaviour, memory, and mental illness, and potentially informing future targeted interventions [96].

Digital technologies, virtual reality, and AI are expanding therapeutic options as adjuncts or alternatives to traditional psychotherapy, including apps, online therapy, therapeutic chats, and therapeutic videogames, which may be particularly useful for young people by matching digital habits, improving adherence, and targeting relevant psychological processes in the context where they matter [97–99] . Digital interventions show promise for anxiety, depression, and ADHD (including an FDA-approved therapeutic videogame). Virtual avatar-based interventions have shown effectiveness for eating disorders by addressing body image distortion in immersive environments [100]. Many technologies still require rigorous validation, emphasising the need for robust studies and quality standards [101].

Lifestyle interventions are increasingly recognised as essential components of integrated mental health care. Evidence supports physical activity and nutrition as relevant adjuncts; physical activity can improve motivation, socialisation, and emotional health [102]. Nutrition and gut microbiota balance may influence mood and cognitive wellbeing [103]. Lifestyle interventions should be designed and supervised by qualified professionals, may also be supported by digital and pharmacological interventions as well, and should be tailored to individual lifestyles and needs.

Treatment selection often remains trial-and-error. AI-enabled computational models using machine learning and causal inference integrate genetic, sociodemographic, brain function, and clinical data to support clinicians, predict response and adverse effects, and personalise dosing through pharmacokinetic data and patterns from electronic health records [73]. Clinical decision support systems are being implemented in some regions to assist prescribing and clinical management [104, 105]. UpToDate also supports rapid access to evidence-based recommendations [106].

### Monitoring and follow‑up

Technological advances support new monitoring and follow-up tools. Sensor-enabled “smart” medications can emit signals upon ingestion to objectively record dosing, supporting adherence and collaborative follow-up [107]. Rapid point-of-care biological tools can detect medication or substance presence in blood in real time, supporting dose adjustment and safety without external laboratories [99].

Mobile apps, wearables, and digital tools enable continuous monitoring of behavioural, emotional, cognitive, and physiological variables (sleep, heart rate, stress), enabling early detection of decompensation, and allow for transdiagnostic clustering across individuals living with psychiatric or neurodegenerative disorders [108]. Sleep alterations can anticipate relapse in bipolar disorder, depression, or psychosis. Platforms such as MONARCA integrate daily self-reports with passively collected smartphone data to support continuous monitoring in bipolar disorder [109].

Telemedicine has improved follow-up by enabling video consultations, supporting adherence, early relapse detection, and access for rural or mobility-limited patients [99, 110–112]. Overall, these innovations support more precise and patient-centred follow-up.

## Challenges

Despite substantial progress, the effective, safe, and equitable implementation of precision medicine in mental health faces persistent challenges across research, ethical and social governance, regulation, and routine clinical practice.

### Research challenges

- *Absence of comprehensive pathophysiological models.* Brain complexity and marked disorder heterogeneity limit the development of valid, reproducible models that can reliably link biological mechanisms to clinical phenotypes. Emerging approaches such as organoids and humanised models represent important advances, but still cannot fully recapitulate the complexity of the human brain.
- *Limited representativeness and diversity.* Many genomic and biomarker findings are derived predominantly from populations of European ancestry, limiting generalisability and clinical applicability across diverse populations. Improving diversity in cohorts and ensuring longitudinal validation are essential for equitable translation into clinical practice.
- *Limited sensitivity, reproducibility, and validation of biomarkers.* Many proposed biomarkers show inconsistent performance across studies and populations, with modest diagnostic accuracy and limited external validation. These limitations, together with high costs and technical requirements, constrain their adoption in real-world clinical settings.
- *Limited integration of biomarkers into conventional diagnostic systems.* Current classifications, such as DSM-5-TR and ICD-11, remain largely descriptive and symptom-based, which limits the systematic incorporation of biological markers and constrains progress toward more objective and mechanism-informed diagnosis and treatment development.
- *Insufficient research funding.* Despite the high clinical, social, and economic burden of mental disorders, investment in mental health research – particularly in precision medicine – remains disproportionately low compared with other major disease areas, limiting innovation, large-scale validation, and translation into clinical practice.
- *Equity* is a central but often under-addressed challenge in precision medicine. Most biomarker, genomic, and digital psychiatry research has been conducted in high-income countries and predominantly in populations of European ancestry, limiting generalisability and potentially exacerbating existing health inequalities. Precision approaches risk benefiting primarily those populations with greater access to advanced diagnostics, digital infrastructures, and specialised care, while underserving socioeconomically disadvantaged groups and low- and middle-income settings. Ensuring equity, therefore, requires deliberate efforts to improve representativeness in research cohorts, adapt tools to diverse cultural and health-system contexts, and align precision psychiatry with principles of Precision Public Health, so that innovation contributes to reducing – rather than widening – mental health disparities.

### Ethical and social challenges

- *Persistent stigma and cultural barriers.* Stigma and cultural factors continue to limit prevention, early diagnosis, and the development and uptake of innovative interventions, undermining help-seeking behaviour, research participation, and equitable access to advances in precision mental health.
- *Risk of misinformation and pseudoscientific narratives.* The spread of misleading or oversimplified information through social media and digital environments can generate confusion, trivialise mental suffering, and erode trust in scientific and clinical sources, thereby distorting public and professional perceptions of advances in precision mental health and hindering their responsible implementation.
- *Technological development outpacing regulation.* Rapid advances in artificial intelligence and digital mental health tools are exceeding existing regulatory frameworks, creating legal uncertainty, variability in standards of care, and potential inequities in access, safety, and accountability.
- *Ethical dilemmas related to neurotechnologies.* Emerging interventions such as neuromodulation, brain–computer interfaces, and optogenetics raise ethical questions regarding autonomy, informed consent, potential behaviour modification, and the boundaries of biomedical intervention, underscoring the need for transparent, proportionate, and participatory governance frameworks.
- *Privacy and confidentiality risks.* The use of genetic, clinical, and behavioural data in artificial intelligence systems raises significant challenges for data protection and confidentiality, including risks of re-identification even after pseudonymisation, which may undermine trust and limit acceptability in clinical and research settings.

### Implementation challenges

- *Limited routine implementation of genetic tools.* The clinical uptake of pharmacogenomics and polygenic risk scores remains limited due to partial risk explanation, ancestry-related performance differences, and the need for specialised interpretation, infrastructure, and clinical workflows to support safe and scalable use.
- *Limited population-level screening strategies for early detection.* Beyond selected high-risk groups or advanced clinical presentations, validated screening approaches for early detection are scarce, highlighting the need for robust early markers, predictive algorithms, and evidence-based frameworks suitable for broader populations.
- *Resistance to technology- and risk-based approaches.* Hesitancy among clinicians and the public may arise from uncertainty about clinical utility, usability concerns, limited training, and fears of stigma or anxiety in asymptomatic populations, underscoring the importance of education, transparency, and ethical communication of risks and benefits.
- *Insufficient multidisciplinary collaborative structures.* Effective implementation of precision mental health is hindered by limited integration across psychiatry, clinical psychology, pharmacology, genetics, neurology, public health, bioinformatics, neuroscience, epidemiology, engineering, and social sciences, despite the inherently cross-disciplinary nature of precision approaches.

## Conclusions and recommendations

Over the past decade, advances in precision medicine have begun to reshape mental health strategies across prevention, diagnosis, treatment, and follow-up, moving care toward more precise, comprehensive, and person-centred models. However, as outlined above, substantial scientific, ethical, regulatory, and implementation barriers continue to limit their safe, equitable, and large-scale integration into routine mental health care.

Ultimately, equitable implementation of precision psychiatry requires not only robust evidence and regulation but also clear and accessible communication that embeds these approaches within routine clinical care.

Addressing these challenges requires coordinated and sustained action involving clinicians, researchers, healthcare managers, policymakers, patients, caregivers, and society at large. In addition, international organisations should promote and coordinate the development of precision psychiatry initiatives. For instance, the ECNP has provided a roadmap for this [1], and the EPA [113] and ECNP have decided to join forces in some of their activities in this area. Precision medicine in mental health should not be understood as a purely technological endeavour, but as a systemic transformation that integrates biological, psychological, social, and environmental dimensions of care within robust ethical and regulatory frameworks.

Based on the challenges identified, the following recommendations are proposed (Table 1):

- *Increase investment in mental health research*, particularly in precision medicine, to levels commensurate with the clinical, social, and economic burden of mental disorders and comparable to other major disease areas such as oncology or cardiovascular medicine.
- *Strengthen translational research pipelines* to improve understanding of brain function and the biological mechanisms underlying mental disorders, facilitating earlier detection, effective prevention, and the development of more precise and personalised therapeutic interventions, while also contributing to stigma reduction through improved biological understanding.
- *Enhance biomarker research and clinical integration* by prioritising sensitivity, reproducibility, and validation across diverse populations and care settings, and by promoting pathways for the responsible incorporation of validated biomarkers into existing diagnostic and clinical decision-making frameworks.
- *Promote dissemination of evidence-based, accessible information* for health professionals, policymakers, patients, and the public to counter misinformation, reduce stigma, and support informed and critical adoption of innovations in precision mental health.
- *Update and harmonise ethical and regulatory frameworks* governing emerging technologies in mental health, including artificial intelligence, digital tools, and neurotechnologies, ensuring robust protections for privacy and confidentiality, clear standards for informed consent, transparency in decision-making, and defined limits for biomedical intervention.
- *Reinforce mental health as a public health priority* through sustained national and regional strategies, coordination structures, and integrated action plans addressing mental health, addictions, suicide prevention, and associated social determinants.
- *Strengthen mental health care services* by supporting the implementation of validated advanced tools, including pharmacogenomics and polygenic risk scores where appropriate, and by scaling up evidence-based early detection, preventive, and predictive programmes within routine care.
- *Expand and strengthen continuing education and training* for mental health professionals in genomics, omics sciences, digital health technologies, data science, and personalised approaches, enabling critical appraisal, appropriate use, and reduced resistance to innovation.
- *Promote multidisciplinary and cross-sector collaboration* across psychiatry, clinical psychology, pharmacology, genetics, neuroscience, public health, bioinformatics, epidemiology, engineering, and social sciences to support integrated approaches spanning prediction, prevention, diagnosis, treatment, and follow-up.

**Table 1.** Key actions for the implementation of precision psychiatry into routine clinical care

Domain	Key actions	Examples of implementation in routine care
Clinical assessment and stratification	Integrate multidimensional assessment beyond categorical diagnosis	Use structured clinical staging, neurocognitive profiling, functional assessment, and relevant biological markers (when validated) to support individualized care planning
Use of biomarkers and omics data	Implement validated biomarkers in a proportionate and clinically meaningful way	Apply pharmacogenetic testing (e.g., CYP2D6/CYP2C19) in selected cases; use inflammatory or metabolic markers to inform treatment selection where evidence supports their utility
Treatment personalisation	Match interventions to individual biological, psychological, and contextual profiles	Tailor pharmacological treatment, psychotherapy, neuromodulation, and lifestyle interventions based on predicted response, tolerability, comorbidity, and patient preferences
Digital tools and monitoring	Incorporate digital technologies for monitoring and follow-up	Use apps, wearables, and telepsychiatry to track symptoms, sleep, adherence, and early warning signs of relapse, ensuring appropriate clinical oversight
Clinical decision support	Support clinicians with evidence-based decision tools	Implement clinical decision support systems integrating clinical data, guidelines, and predictive models to reduce trial-and-error prescribing
Workforce training and competencies	Strengthen professional training in precision approaches	Provide continuing education in genomics, data interpretation, digital mental health, and the ethical use of AI for mental health professionals
Ethical, legal, and social governance	Ensure responsible and transparent use of data and technologies	Apply robust informed consent, data protection, explainability of AI tools, and safeguards against stigma or misuse of predictive information
Health system organization	Adapt services to support precision-based care models	Develop multidisciplinary teams, integrate mental health with primary and specialty care, and ensure equitable access to validated precision tools
Equity and representativeness	Reduce disparities in access and applicability	Promote inclusive implementation strategies that account for cultural, socioeconomic, and population diversity across health systems
Evaluation and continuous improvement	Monitor outcomes and refine implementation	Use real-world data, quality indicators, and patient-reported outcomes to iteratively assess effectiveness, safety, and acceptability

In conclusion, precision medicine offers a transformative opportunity to improve mental health care, but its potential will only be realised through deliberate, coordinated, and ethically grounded implementation. Aligning scientific innovation with clinical relevance, social responsibility, and health system readiness is essential to ensure that advances in precision mental health translate into tangible and equitable benefits for individuals and populations.
