# Precision medicine in mental health: applications, challenges, and recommendations – CORRIGENDUM

**DOI:** 10.1192/j.eurpsy.2026.12230

**Published:** 2026-06-29

**Authors:** Celso Arango, Eduard Vieta, Lourdes Fañañás, Philippe Courtet, Livia De Picker, Martien J.H. Kas, Peter Kéri, Pavel Mohr, Inez Myin-Germeys, Brenda W.J.H. Penninx, Andreas Reif, Martina Rojnic Kuzman, Marion Leboyer, Andrea Fiorillo, Ana Catalán

This article was originally published with a error in the author list. The name of Ana Catalán was misspelt as Ana Cataln. This has now been corrected and the online article and PDF have been updated.

The authors apologise for this error.
